# Clinical course of patients with bloodstream infections enrolled in the BALANCE clinical trial

**DOI:** 10.1093/jac/dkaf294

**Published:** 2025-08-04

**Authors:** Sean W X Ong, Ruxandra Pinto, Asgar Rishu, Steven Y C Tong, Robert A Fowler, Nick Daneman

**Affiliations:** Institute of Health Policy, Management and Evaluation, University of Toronto, Toronto, Canada; Department of Infectious Diseases, University of Melbourne, Peter Doherty Institute for Infection and Immunity, Melbourne, Victoria, Australia; Sunnybrook Health Sciences Centre, Toronto, Canada; Victorian Infectious Diseases Service, Royal Melbourne Hospital, Peter Doherty Institute for Infection and Immunity, Melbourne, Australia; Institute of Health Policy, Management and Evaluation, University of Toronto, Toronto, Canada; Sunnybrook Health Sciences Centre, Toronto, Canada; Sunnybrook Health Sciences Centre, Toronto, Canada; Department of Infectious Diseases, University of Melbourne, Peter Doherty Institute for Infection and Immunity, Melbourne, Victoria, Australia; Victorian Infectious Diseases Service, Royal Melbourne Hospital, Peter Doherty Institute for Infection and Immunity, Melbourne, Australia; Institute of Health Policy, Management and Evaluation, University of Toronto, Toronto, Canada; Sunnybrook Health Sciences Centre, Toronto, Canada; Institute of Health Policy, Management and Evaluation, University of Toronto, Toronto, Canada; Sunnybrook Health Sciences Centre, Toronto, Canada

## Abstract

**Objectives:**

There is a lack of data describing the longitudinal clinical trajectories of vital signs and laboratory tests in patients with bloodstream infection (BSI). The BALANCE trial, which randomly assigned patients with BSI to receive 7 or 14 days of antibiotic treatment, provided rich daily data to describe these trajectories.

**Methods:**

As part of the BALANCE trial, we collected several daily parameters (temperature, heart rate, mean arterial pressure, systolic blood pressure, respiratory rate, WBC count, C-reactive protein, platelet count and SOFA score) until Day 14 of illness, discharge or death. In this *post hoc* descriptive sub-study, we described trajectories of these parameters, stratified by treatment group allocation and by the primary outcome of 90-day all-cause mortality.

**Results:**

Among 3608 patients included, median age was 70 years and 46.7% were female. At enrolment, 55.0% were admitted in the ICU and 21.2% required mechanical ventilation. Longitudinal trajectories of vital signs, laboratory tests and SOFA scores were almost identical comparing the two treatment groups, including from Day 7 after treatment divergence. These trajectories were markedly different when comparing survivors (3034 patients; 84.7%) and non-survivors (547 patients; 15.3%), with non-survivors demonstrating a slower recovery course throughout the 14-day period.

**Conclusions:**

Among hospitalized patients with BSI, recovery trajectories were similar in patients assigned to 7- versus 14-day antibiotic treatment durations but were different comparing survivors versus non-survivors. These data could be used to inform daily clinical management, formulate predictive risk scores or clinical decision rules, and guide future research into individualized therapeutic strategies.

## Introduction

Bloodstream infections (BSI) are a common problem encountered in the inpatient setting and are associated with significant mortality and morbidity.^1^ Optimizing the clinical management of BSI may be an important way to improve patient outcomes. An integral part of this is the daily assessment of patients, which assists clinicians in determining whether patients are responding favourably to treatment or if further diagnostic or therapeutic interventions are required.

However, there is a lack of data describing the longitudinal clinical trajectories of vital signs and laboratory parameters for these patients. Previous studies have demonstrated that there is wide variability in these trajectories, but many have focused on infectious syndromes other than BSI, had modest sample sizes, frequently been from a single centre or not had the opportunity to report detailed daily data.^2–9^

The recently published BALANCE (Bacteremia Antibiotic Length Actually Needed for Clinical Effectiveness) clinical trial provides an opportunity to characterize the temporal course of 3608 patients hospitalized with BSI, recruited across 74 centres in 7 countries.^10^ The BALANCE trial randomized patients to receive 7 versus 14 days of antibiotic treatment. It demonstrated non-inferiority for the primary outcome of 90-day all-cause mortality with 7-day treatment. The aims of this *post hoc* descriptive sub-study were to describe the daily course of vital signs and laboratory tests among hospitalized patients with BSI, stratified by treatment allocation (7 versus 14 days of antibiotic treatment) and 90-day survival status.

## Methods

Data were obtained from the BALANCE trial, which enrolled patients between October 2014 and June 2023. Patients with BSI, defined as having a positive blood culture with pathogenic bacteria, were included. Key exclusion criteria were infection with *Staphylococcus aureus* or *Staphylococcus lugdunensis*, *Candida* or fungal species, a single positive culture with a common contaminant organism, severe immunocompromise or a well-defined requirement for prolonged treatment (e.g. endocarditis). The primary outcome of the trial was 90-day all-cause mortality. Full trial details are available in the trial protocol and primary manuscript.^10,11^ As a pragmatic clinical trial, laboratory procedures were not standardized, and biochemical and microbiological methods were based on standard procedures at each study site.

The following eight parameters of interest were captured and described: temperature, heart rate, mean arterial pressure (MAP), systolic blood pressure (SBP), respiratory rate (RR), WBC count, C-reactive protein (CRP) and platelet count. These parameters were chosen as they are readily available and frequently monitored in clinical practice to assess clinical response in patients with BSI. In addition, the SOFA score along with its six specific organ score components (respiratory, cardiovascular, hepatic, coagulation, renal and neurological) are also reported.^12^ Clinical data were obtained by research staff at each site via manual chart review on each hospitalization day, until Day 14 of illness, discharge or death, whichever was earliest. For vital sign parameters, the most extreme abnormal daily measurement of any given day was recorded. This included highest values for temperature, RR, WBC count and CRP, and lowest values for platelet count, MAP and SBP. The timings of laboratory measurements were not protocolized in the trial and were decided by individual clinicians. Therefore, not all laboratory tests were available for each day, and we only included available observations for each test. Missing WBC, CRP and platelet counts were not imputed.

Missing vital sign parameters (HR, RR, SBP and MAP) were imputed with the previous or next value if available or the average of both values if both were available. Only the first value was imputed with the previous day's value if data from multiple consecutive days were missing. Missing SOFA score components were also imputed to calculate SOFA scores for as many patients as feasible (see Supplementary Methods, available as Supplementary data at *JAC* Online, for further details).

For each parameter, we plotted mean values on each day of illness with CIs around the mean for the entire trial cohort stratified by treatment group allocation (7 versus 14 days of antibiotics) to assess any differences in the time course of recovery based on treatment group. We also plotted the same graphs, but with the cohort stratified by 90-day survival status, as well as ICU status at enrolment. In addition, for each parameter, we calculated the proportion of patients with values within the specified normal range for that parameter on each day of illness, again stratified by treatment group allocation and outcome status. Days of illness were counted in reference to Day 0 being the onset of BSI, as defined as the date of collection of the first positive blood culture. As the primary aim of this study was descriptive, we did not conduct any inferential testing or statistical tests of association. We also did not account or adjust for the possibility that repeated measures may represent more severe or abnormal values (patients with more severe illness or those with initial abnormalities may be more likely to have repeat measurements). Data management and visualization were conducted in R version 4.4.0.

## Results

### Study cohort

**Figure 1.**
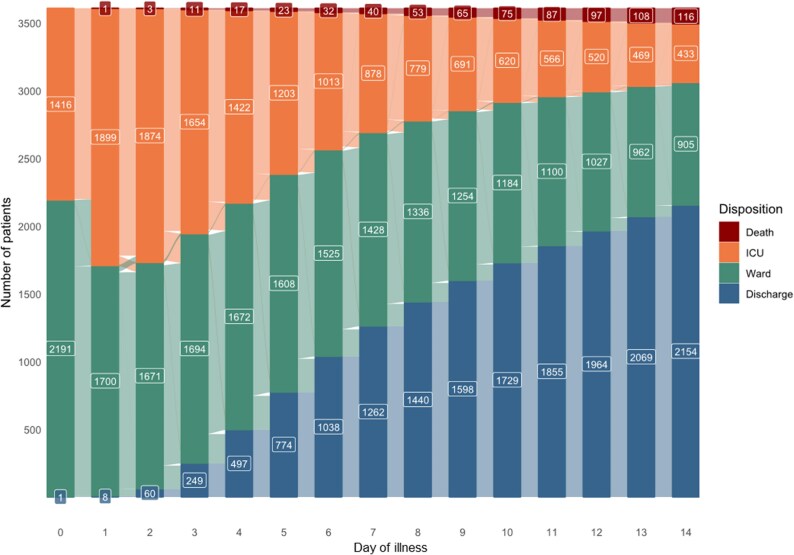
Patient status of the entire study cohort on each day of illness. This alluvial chart depicts the flow of patients in the study based on their status on each day of illness. Days of illness were counted in reference to the date of the index positive blood culture collection (Day 0). Stacked bar charts depict the number of patients who died (dark red), were in the ICU (orange), on the ward (green), or were discharged alive (blue). Flows between each bar show the number of patients moving between each state (e.g. from ward to ICU, ICU to ward, ward to discharge, etc.).

The demographics and detailed baseline characteristics of the BALANCE trial cohort have been previously reported.^10^ In brief, among 3608 patients, median age was 70 years and 46.7% were female, 55.0% were admitted in the ICU and 21.2% required mechanical ventilation at the time of enrolment. The flow of all the patients in the cohort through various states (ICU, ward, discharge or death) per day is summarized in Figure 1. The 90-day all-cause mortality was 15.3% (547 of 3581 patients with outcome data available). Baseline characteristics of the cohort, stratified by 90-day mortality status, are summarized in Table S2. Patients who died within 90 days were more likely to be male, of older age, have more comorbidity, have more severe disease at baseline, non-urinary source of infection and non-*Enterobacterales* infection.

### Laboratory test values

**Figure 2.**
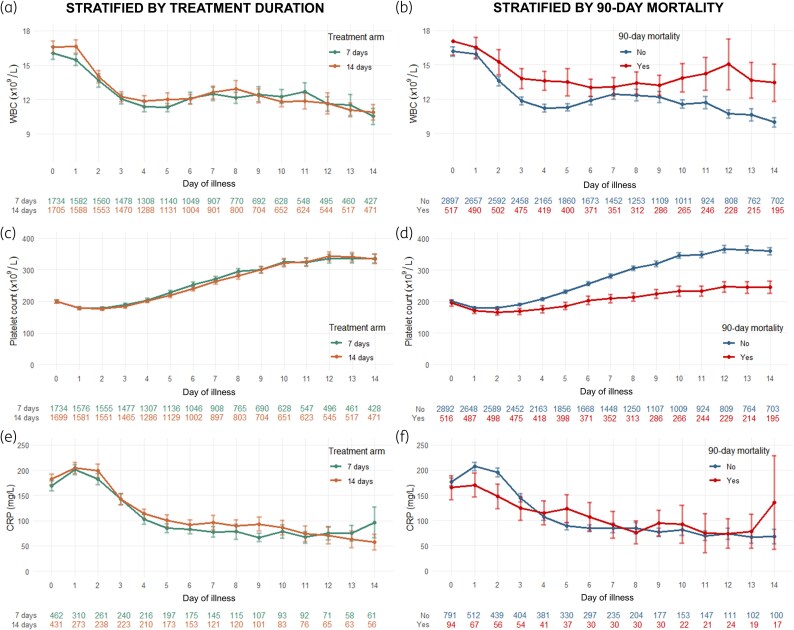
Mean WBC, platelet and CRP values by each day of illness, stratified by treatment allocation group and outcome status. Points depict mean values for each day of illness for each subgroup, while error bars depict CIs around the mean. Panels on the left [(a), (c) and (e)] show the subgroup stratified by treatment allocation group and panels on the right [(b), (d) and (f)] show the cohort stratified by primary outcome (90-day mortality). Corresponding numbers below each graph show the number of patients with available observations per subgroup for each day (e.g. on Day 14 there were 427 patients in the 7-day arm and 471 patients in the 14-day arm who had available WBC values). Days of illness were counted in reference to the date of the index positive blood culture collection (Day 0).

The longitudinal trajectories of mean WBC count, CRP level, and platelet count for the entire cohort are shown in Figure 2. Trajectories were similar comparing the 7- and 14-day treatment groups but differed between the survivor versus non-survivor groups. Mean WBC count was higher at each day of illness in the non-survivor group (Figure 2b). The slope of CRP decline from Days 1 to 5 appeared to be rapid in the survivor group (Figure 2f), while platelet count appeared to recover more slowly in the non-survivor group (Figure 2d). These trends were similar in the subgroup of patients with abnormal baseline values (i.e. excluding patients with normal values of each laboratory test on Day 0 of illness) (Figure S1).

### Vital signs

**Figure 3.**
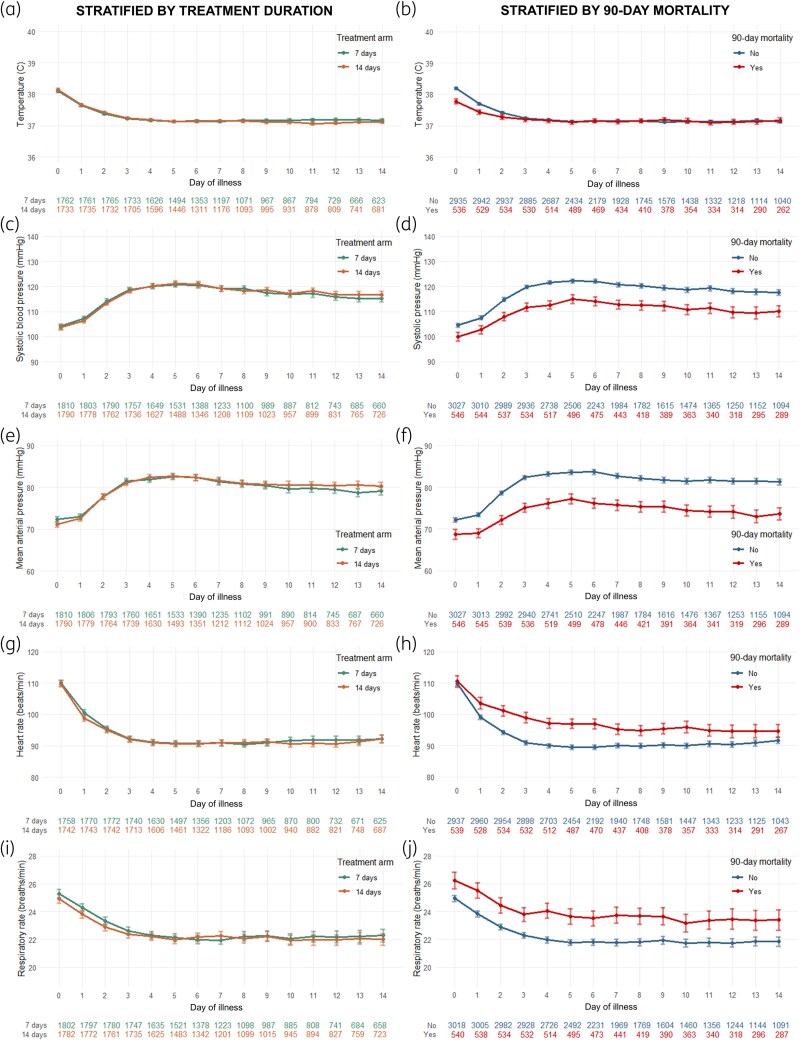
Mean values of five vital signs by each day of illness, stratified by treatment allocation group and outcome status. Points depict mean values for each day of illness for each subgroup, while error bars depict CIs around the mean. Panels on the left [(a), (c), (e), (g) and (i)] show the subgroup stratified by treatment allocation group and panels on the right [(b), (d), (f), (h) and (j)] show the cohort stratified by primary outcome (90-day mortality). Corresponding numbers below each graph show the number of patients with available observations per subgroup for each day (e.g. on Day 14 there were 623 patients in the 7-day arm and 681 patients in the 14-day arm who had available temperature readings). Days of illness were counted in reference to the date of the index positive blood culture collection (Day 0).

The mean values of the five vital signs per day are shown in Figure 3. Trajectories were almost superimposable when stratified by treatment group, but diverged when stratified by mortality outcome. Temperature was higher in the first 3 days in the group who survived but converged after Day 3 (Figure 3b). In contrast, SBP, MAP, HR and RR were more abnormal throughout the entire course of illness among non-survivors compared with survivors (Figure 3d, f, h and j). The same recovery trends were observed for both the composite SOFA score and individual organ-specific SOFA score components (Figure S2). Patients who were in ICU at enrolment versus those who were not had similar trajectories of clinical parameters, although there was divergence in mean values of most parameters (Figure S3).

### Proportion of patients with normal parameters

The proportion of patients with parameters within the normal range for temperature, HR, MAP, WBC and CRP per day of illness, both for the entire cohort and for the subgroup with abnormal values at baseline, is shown in Figure S4. Approximately 25% of patients had fever beyond 3 days. Characteristics of patients with and without persistent fever beyond 3 days are compared in Table S3. Recovery for MAP and HR similarly occurred in the first 3 days, beyond which approximately 33% and 25% of the cohort had ongoing derangements, respectively. WBC remained abnormal for almost half the cohort up to 14 days. CRP normalization was even slower, with the majority of patients (approximately 75%) having persistently elevated CRP up to 14 days.

## Discussion

In this descriptive *post hoc* sub-study of the BALANCE trial, we report the longitudinal trajectory of a range of vital signs and laboratory parameters for hospitalized patients with BSI, providing a detailed 2-week description of the disease course. The strengths of our study are its large sample size, rich daily data, and inclusion of a large proportion of critically ill patients. The international multicentre nature of the study makes it likely that our findings are generalizable to other high-income healthcare settings.

Our results can be used to contextualize the interpretation of clinical parameters and laboratory tests during the daily evaluation of patients with BSI. For example, we show that one can expect about 50% of patients with fever at baseline to defervesce by Day 1 and 75% by Day 3. On the flipside, up to 25% of patients have fever beyond 3 days. Other studies have reported similar findings, and similarly persistent fever was not associated with differences in clinical outcomes of clinical cure or mortality.^2,13,14^ Time to defervescence should thus not be considered a reason to extend duration of antibiotics, in the absence of other convincing indications. Similar recovery trajectories can be observed for other physiologic derangements such as tachycardia or hypotension. In contrast, elevated inflammatory marker levels will take longer to resolve—less than half and less than one-quarter of patients with elevated WBC or CRP at baseline will have these normalize within the first week.

We observed almost identical recovery trajectories comparing patients who were randomly assigned to receive 7 versus 14 days of antibiotics, even after treatment divergence beyond Day 7. This reinforces the findings from the primary BALANCE analysis that a shorter 7-day course of antibiotic treatment is sufficient for treatment of most patients with BSI, not only from a mortality standpoint but also in terms of disease recovery parameters in the first 14 days.

In contrast, there was divergence in many of these parameters, including WBC count, platelet count, MAP, SBP, HR and RR, between the subgroup who died within 90 days and the subgroup who did not. These data can be used in future studies to inform construction of clinical criteria or decision rules to predict risk, evaluate treatment response and individualize treatment durations. For example, Rac *et al*. developed criteria to define early clinical failure in Gram-negative BSI, incorporating SBP, HR, RR, altered mental status and WBC count, and found that these criteria could predict 28-day mortality and prolonged hospitalization.^15^ We have used these data to validate this risk score in a separate analysis and found that their criteria could reliably predict 90-day mortality in our cohort.^16^

There appeared to be no difference in the temperature trajectories after Day 3 comparing patients who died within 90 days versus those who did not. Those who did not die within 90 days appeared to have a higher absolute temperature and a higher proportion of fever at baseline, which was consistent with previous data in the literature.^17^ Higher temperatures have been previously associated with favourable outcomes in patients with infection.^18^ Whether this is due to a more robust inflammatory response signifying underlying stronger host immunity, or a beneficial effect of fever *per se*, is unclear. The impact of pharmacological or non-pharmacological interventions to treat fever on clinical outcomes is controversial, and multiple clinical trials have not demonstrated a benefit with antipyretic therapies.^19,20^

The slope of CRP decline appeared to differ between the survivor versus non-survivor subgroups, with a more rapid decline among patients who survived 90 days. Previous small studies have shown that the rate of CRP decline can be predictive of clinical outcomes in multiple infectious syndromes including BSI,^21–23^ highlighting the need for further research to evaluate the utility of this biomarker in predicting risk or guiding treatment decisions. However, a clinical stopping rule relying on CRP or WBC normalization may lead to unnecessary treatment prolongation, since these values may remain abnormal up to at least 14 days, even though the BALANCE trial demonstrated non-inferiority of a shorter 7-day antibiotic duration.

There are several limitations to our study. First, this study was primarily descriptive, and we did not conduct formal statistical testing to relate longitudinal trajectories of each parameter to prediction of clinical outcomes. We opted not to do this since the frequency of these measurements was not protocolized given the pragmatic nature of this trial. Left to individual clinicians’ discretion, it is thus likely that patients with more severe illness had a higher frequency of these measurements, which could result in selection bias. Those with initial abnormalities may also be more likely to have repeated tests as opposed to those with normal values at baseline. To mitigate this latter issue, we plotted the same graphs in the subset of the cohort with abnormal values of each laboratory test at baseline. Second, the duration of data collection was to Day 14 of illness, discharge or death, whichever was earliest. As the day of discharge was clinician determined and also not protocolized, it is likely that those who were discharged alive before 14 days had milder illness (and less likely to have concerningly abnormal vital signs or laboratory parameters), and those who remained in hospital (and hence had available data) had more severe illness. Patients in the 14-day treatment group may have also been preferentially kept in hospital for a longer duration. Indeed, there were more observations for each parameter for each day in the 14-day group after Day 7 of illness. This could have resulted in differences between the 7- and 14-day treatment groups after Day 7 and may explain some of the minor divergences in the longitudinal trajectories between the two groups. Yet, median hospital length of stay was only 1 day longer for those in the 14-day treatment arm in the BALANCE RCT; therefore, the influence of a differential length of stay due to a beneficial outcome in the 7-day treatment arm, or due to clinician decision making, is likely small. Third, we did not collect data on some other variables that may affect some of these longitudinal trajectories. For example, there were no data on temperature management interventions (e.g. acetaminophen, physical cooling measures). Blood pressure and RR may also be tightly controlled in the ICU, though these interventions are reflected in the SOFA score (e.g. inotropic and ventilatory requirements), which may thus better reflect the recovery trajectories among ICU patients. We also did not account for adequacy of antibiotic treatment (e.g. isolate susceptibility versus antibiotic spectrum mismatch) as a potential reason for persistent fever or delayed recovery. Fourth, we had limited data on other potentially prognostically important biomarkers such as procalcitonin, which was not commonly used in the settings where the BALANCE trial was conducted. Finally, while 55% of patients were recruited from the ICU, our study cohort has relatively lower severity of illness compared with other observational cohorts of BSI in ICU patients,^24,25^ which may reflect a degree of selection bias in BALANCE; given that the intervention arms only diverged at 7 days, the trial was not intended to enrol moribund patients or those not expected to survive to Day 7. Other studies have shown that patients enrolled in RCTs may differ in comorbidity profile and disease severity from the general patient population, which may limit generalizability of our findings.^26,27^

### Conclusion

We describe the time course of several key vital signs and laboratory tests in patients with BSI in the first 14 days of their illness. Recovery trajectories were similar in patients assigned to 7- versus 14-day antibiotic treatment durations but were different comparing survivors versus non-survivors. These data could be used to inform daily clinical management, formulate predictive risk scores or clinical decision rules and guide future research into individualized therapeutic strategies for BSI.

## Supplementary Material

dkaf294_Supplementary_Data

## Supplementary data

Figures S1–S4 and Tables S1–S3 are available as Supplementary data at *JAC*  Online.
