# Supplementary material for: Clinical course of patients with bloodstream infections enrolled in the BALANCE clinical trial
Source: J Antimicrob Chemother. 2025 Aug 4;80(10):2752–8. doi: 10.1093/jac/dkaf294 (PMC12494135; doi:10.1093/jac/dkaf294)
Supplement: dkaf294_Supplementary_Data [file dkaf294_supplementary_data.docx]

**Supplementary Appendix – Ong et al., Clinical course of patients with bloodstream infections enrolled in the BALANCE clinical trial.**

**Contents:**

1. Supplementary Methods
2. Table S1: Full list of BALANCE trial consortium members
3. Table S2: Baseline characteristics of the study cohort, stratified by 90-day mortality
4. Table S3: Baseline characteristics of the study cohort, stratified by presence of persistent fever (>72 hours)
5. Figure S1: Mean white blood cell count, platelet, and C-reactive protein count on each day of illness, excluding patients with normal baseline values.
6. Figure S2: Mean composite SOFA score and individual organ-specific SOFA score components on each day of illness.
7. Figure S3: Mean values of eight clinical parameters by each day of illness, stratified by intensive care status at enrolment
8. Figure S4: Proportion of participants with normal temperature, heart rate, mean arterial pressure, white blood cell count, and C-reactive protein on each day of illness.
9. **Supplementary Methods**

*Imputation of missing SOFA score components*

Missing SOFA score components were imputed using the following rules:

Respiratory SOFA score:

- If FiO_2_ was missing and volume of supplemental oxygen was available in litres/min, this was converted to FiO_2_ using a conversion chart
- A maximum of one consecutive PaO_2_/FiO_2_ ratio was imputed using previous or next day values if available, or the mean of both if both were available
- If PaO_2_/FiO_2_ ratio remained missing, the respiratory SOFA score was imputed as 0
- If PaO_2_/FiO_2_ ratio was between 0-300 and the mechanical ventilation status was missing, the respiratory SOFA score was imputed as 0

Coagulation SOFA score:

- A maximum of one consecutive platelet count was imputed with previous or next day values if available, or the mean of both if both were available
- If platelet count remained missing, the coagulation SOFA score was imputed as 0

Liver SOFA score:

- As bilirubin is typically measured less frequently, the bilirubin count was imputed for up to 3 consecutive days if missing using the prior value, subsequent value, or mean of both if both were available
- If after imputation bilirubin level remained missing, the liver SOFA score was imputed as 0

Cardiovascular SOFA score:

- Missing MAP values were imputed as described
- Vasopressor requirement as imputed as “no” if patients were not in the ICU (as vasopressors could only be administered in the ICU
- If after imputation, MAP or vasopressor requirement was missing, the cardiovascular SOFA score was imputed as 0

Neurological SOFA score:

- A maximum of one consecutive missing Glasgow Coma Scale (GCS) values was imputed with previous or next day values if available, or the lower of both if both previous and next values were available
- If GCS remained missing, the neurological SOFA score was imputed as 0

Renal SOFA score:

- A maximum of one consecutive creatinine value was imputed with previous or next day values if available, or the mean of both if both previous and next values were available
- If urine output was missing but creatinine was available, then the renal SOFA score was determined using creatinine level only; and vice versa
- If urine was collected for <24h, it was considered missing
- Urine output was not imputed
- If both creatinine and urine output were missing, the renal SOFA score was imputed as 0

1. **Table S1: Full list of BALANCE trial consortium members.**

| **Name** | **Affiliation** |
| --- | --- |
| Nick Daneman | Division of Infectious Diseases, Department of Medicine, Sunnybrook Health Sciences Centre, University of Toronto, Toronto, Canada |
| Asgar Rishu | Sunnybrook Research Institute, Sunnybrook Health Sciences Centre, University of Toronto, Ontario, Canada |
| Ruxandra Pinto | Sunnybrook Health Sciences Centre, Toronto, Ontario, Canada |
| Benjamin Rogers | Monash University, Clayton, Melbourne, Australia |
| Yahya Shehabi | Department of Intensive Care, Monash Medical Centre, Melbourne, VIC, Australia |
| Rachael Parke | Cardiothoracic and Vascular Intensive Care Unit, Auckland City Hospital, Auckland, New Zealand |
| Deborah J Cook | Department of Medicine, Faculty of Health Sciences, McMaster University, Hamilton, Ontario, Canada |
| Yaseen Arabi | Intensive Care Department, College of Medicine, King Saud Bin Abdulaziz University for Health Sciences, King Abdullah International Medical Research Center, Riyadh, Saudi Arabia |
| John Muscedere | Department of Critical Care Medicine, Queen's University, Kingston, Ontario, Canada |
| Steven Reynolds | Department of Critical Care Medicine, Royal Columbian Hospital, University of British Columbia, Vancouver, British Columbia, Canada |
| Richard Hall | Critical Care Medicine, Capital District Health Authority, Dalhousie University, Canada |
| Dhiraj Bhatia Dwivedi | Monash Medical Centre, 246 Clayton Road, Clayton, Vic 3168, Australia |
| Colin McArthur | Critical Care Medicine, Auckland City Hospital, New Zealand |
| Shay McGuinness | Cardiothoracic and Vascular Intensive Care Unit, Auckland City Hospital, Auckland, New Zealand |
| Dafna Yahav | Infectious Diseases Unit, Sheba Medical Center, Ramat-Gan, Israel and Faculty of medicine, RamatAviv, Tel-Aviv, Israel |
| Bryan Coburn | Infectious Diseases, University Health Network, University of Toronto, Canada |
| Anna Geagea | Critical Care Medicine, North York General Hospital, Canada |
| Pavani Das | Infectious Diseases, North York General Hospital, Ontario, Canada |
| Phillip Shin | Critical Care Medicine, North York General Hospital, Ontario, Canada |
| Michael Detsky | Critical Care Medicine, Mount Sinai Hospital, Unity Health Toronto, Canada |
| Andrew Morris | Department of Medicine, University of Toronto, Canada |
| Michael Fralick | Sinai Health, Division of General Internal Medicine, Toronto, Ontario, Canada |
| Jeff Powis | Infectious Diseases, Michael Garron Hospital, University of Toronto, Canada |
| Christopher Kandel | Infectious Diseases, Michael Garron Hospital, Canada |
| Wendy Sligl | Critical Care Medicine and Infectious Diseases, University of Alberta, Canada |
| Sean M Bagshaw | Department of Critical Care Medicine, Faculty of Medicine and Dentistry, University of Alberta and Alberta Health Services, Canada |
| Nishma Singhal | Department of Medicine, Hamilton Health Sciences, McMaster University, Canada |
| Emilie Belley-Cote | Department of Anaesthesia, Hamilton General Hospital, McMaster University, Hamilton, Canada |
| Richard Whitlock | Faculty of Health Sciences, Hamilton General Hospital, McMaster University, Hamilton, Canada |
| Kosar Khwaja | Departments of Surgery and Critical Care, McGill University Health Center, Canada |
| Susan Morpeth | Departments of Infectious Diseases and Pathology, Middlemore hospital, Middlemore hospital and the University of Auckland, New Zealand |
| Alex Kazemi | Organ Donation New Zealand, New Zealand Blood Service, New Zealand |
| Tony Williams | Organ Donation New Zealand, New Zealand Blood Service, New Zealand |
| Derek MacFadden | Division of Infectious Diseases, The Ottawa Hospital, The Ottawa Hospital Research Institute, Canada |
| Lauralyn McIntyre | Department of Medicine, Ottawa Hospital, University of Ottawa, Ottawa, Canada |
| Jennifer LY Tsang | Niagara Health Knowledge Institute, Niagara Health, Canada |
| Francois Lamontagne | Department of Medicine, Université de Sherbrooke, Canada |
| Alex Carignan | Department of Microbiology and Infectious Diseases, Université de Sherbrooke, Canada |
| John Marshall | Surgery and Critical Care Medicine, Unity Health Toronto; University of Toronto, Canada |
| Jan O Friedrich | Critical Care and Medicine, Unity Health Toronto, St. Michael's Hospital, University of Toronto, Canada |
| Rob Cirone | Critical Care Medicine, Unity Health Toronto, Canada |
| Mark Downing | Department of Medicine, Unity Health Toronto, Canada |
| Christopher Graham | Department of Medicine, Infectious Diseases, Trillium Health Partners, University of Toronto, Canada |
| Joshua Davis | School of Medicine and Public Health, University of Newcastle, Australia |
| Erick Duan | Assistant Professor, Division of Critical Care, Department of Medicine, McMaster University, Canada |
| John Neary | Medicine, St. Joseph's Healthcare Hamilton, McMaster University, Canada |
| Gerald Evans | Department of Medicine (Infectious Diseases), Queen's University, Canada |
| Basem Alraddadi | Department of Medicine, King Faisal Specialist Hospital and Research Centre, Al Faisal University, Jeddah Saudi Arabia |
| Sameera Aljohani | Department of Pathology & Laboratory Medicine, King Saud Bin Abdulaziz University for Health Sciences, King Abdullah International Medical Research Center, Riyadh, Saudi Arabia |
| Claudio Martin | Department of Medicine, University of Western Ontario, London, ON, Canada |
| Sameer Elsayed | Department of Medicine, Division of Infectious Diseases, London Health Sciences Centre, London, Ontario, Canada |
| Ian Ball | Department of Medicine, Western University, Canada |
| François Lauzier | Department of Medicine, Université Laval, Canada |
| Alexis F. Turgeon | Department of Anesthesiology and Critical Care Medicine, Faculty of Medicine, Université Laval, Quebec City, Canada.  Population Health and Optimal Health Practice Research Unit, Centre Hospitalier Universitaire de Québec - Université Laval Research Center, Québec, Canada |
| Henry Thomas Stelfox | Department of Critical Care, University of Calgary Cumming School of Medicine, Calgary, Alberta, Canada |
| John Conly | Department of Medicine, University of Calgary and Alberta Health Services (Calgary), Canada |
| Todd C. Lee | Clinical Practice Assessment Unit, McGill University, Montreal, canada |
| Emily G. McDonald | Division of General Internal Medicine; Department of Medicine, McGill University Health Centre, Canada |
| Richard Sullivan | Department Infectious Diseases, St George Hospital, School of Clinical Medicine, UNSW Medicine and Health, Australia |
| Jennifer Grant | Divisions of Infectious Diseases and Medical Microbiology, University of British Columbia, Canada |
| Ilya Kagan | Intensive care unit, Rabin medical centers, Tel Aviv university, Israel |
| Paul Young | Intensive Care Research Programme, Medical Research Institute of New Zealand, New Zealand |
| Cassie Lawrence | Medical Research Institute of New Zealand, Wellington, New Zealand |
| Kevin O'Callaghan | Department of Infectious Diseases, Redcliffe Hospital, Australia |
| Matthew Eustace | Infectious Diseases, Redcliffe Hospital, University of Queensland, Australia |
| Keat Choong | Infectious Diseases, Sunshine Coast University Hospital, Sunshine Coast University Hospital, Australia |
| Pierre Aslanian | Medicine, Centre Hospitalier de l’Université de Montréal, Université de Montréal, Canada |
| Ulrike Buehner | Department of Anaesthesia, Rotorua Hospital, Rotorua, New Zealand |
| Tom Havey | Infectious Diseases, William Osler Health System, Canada |
| Alexandra Binnie | Critical Care Medicine, William Osler Health System, Canada |
| Josef Prazak | Department of Intensive Care Medicine, Bern University Hospital, University of Bern, Bern, Switzerland |
| Brenda Reeve | Brantford General Hospital, McMaster University, Brantford, Canada |
| Edward Litton | Intensive Care Unit, Fiona Stanley Hospital, School of Medicine, University of Western Australia, Australia |
| Sylvain Lother | Department of Medicine, University of Manitoba, Canada |
| Anand Kumar | Division of Critical Care Medicine and Infectious Diseases, Health Sciences Centre, University of Manitoba, Winnipeg, Canada |
| Ryan Zarychanski | Department of Internal Medicine, Rady Faculty of Health Sciences, University of Manitoba, Canada |
| Tomer Hoffman | Infectious diseases unit, Sheba medical center, Israel |
| David L. Paterson | Infectious Diseases Unit, Royal Brisbane and Women's Hospital, Brisbane, Australia |
| Peter Daley | Infectious Diseases, Memorial University, NL, Canada |
| Robert J Commons | General and Subspecialty Medicine, Grampians Health Ballarat, Ballarat, Victoria, Australia |
| Emmanuel Charbonney | Service des soins intensifs, Centre Hospitalier de l'Université de Montréal (CHUM), Montreal, Canada |
| Jean-Francois Naud | Critical Care Medicine, CIUSSS MCQ CHAUR, University of Montreal, Canada |
| Sally Roberts | Clinical Microbiology and Infection Prevention and Control, Auckland Hospital, Auckland, New Zealand |
| Ravindranath Tiruvoipati | Department of Intensive Care Medicine, Frankston Hospital, Frankston, Victoria, Australia |
| Sachin Gupta | Department of Intensive Care Adjunct Senior Lecturer, Monash University, Victoria, Australia |
| Gordon Wood | Department of Critical Care, Island Health Authority, Royal Jubilee Hospital, British Columbia, Canada |
| Omar Shum | Infectious Diseases, The Wollongong Hospital, Australia |
| Spiros Miyakis | Infectious Diseases, Wollongong Hospital, Graduate School of Medicine, University of Wollongong, Australia |
| Peter Dodek | Department of Medicine, St. Paul's Hospital, University of British Columbia, Canada |
| Clement Kwok | Infectious Diseases, Richmond Hospital, Canada |
| Linda R Taggart | Division of Infectious Diseases, Department of Medicine, St. Michael’s Hospital, Unity Health Toronto, Toronto, Ontario, Canada |
| Stephanie Smith | Department of Medicine, University of Alberta, Edmonton, Alberta, Canada |
| Karen Doucette | Division of Infectious Diseases, University of Alberta, Edmonton, Alberta, Canada |
| Robert Fowler | Interdepartmental Division of Critical Care Medicine, Department of Medicine, Sunnybrook Health Sciences Centre, University of Toronto, Ontario, Canada |

1. **Table S2: Baseline characteristics of the study cohort, stratified by 90-day mortality**

| **Characteristic** | **Overall**  **(n=3608)** | **Survivors**  **(n=3034)** | **Non-survivors**  **(n=547)** | ***p*-value** |
| --- | --- | --- | --- | --- |
| Male sex | 1922 (53.3) | 1586 (52.3) | 315 (57.6) | 0.025 |
| Age, years | 70 (59–80) | 69 (58–79) | 74 (63–82) | <0.001 |
| Baseline SOFA score | 4 (2–8) | 4 (2–8) | 6 (3–10) | <0.001 |
| ICU admission at enrolment | 1986 (55.0) | 1620 (53.4) | 361 (66.0) | <0.001 |
| Mechanical ventilation at baseline | 766 (21.2) | 604 (19.9) | 161 (29.4) | <0.001 |
| Diabetes mellitus | 1148 (31.8) | 958 (32.0) | 184 (33.9) | 0.40 |
| Solid organ cancer | 782 (21.7) | 594 (19.8) | 187 (34.5) | <0.001 |
| Obesity | 655 (18.2) | 580 (19.4) | 77 (14.2) | 0.005 |
| Arrhythmia | 540 (15.0) | 429 (14.3) | 109 (20.1) | 0.001 |
| Immunosuppression | 299 (8.3) | 233 (7.8) | 66 (12.2) | 0.001 |
| Chronic obstructive pulmonary disease | 393 (10.9) | 307 (10.3) | 83 (15.3) | 0.001 |
| Renal insufficiency | 425 (11.8) | 319 (10.7) | 105 (19.4) | <0.001 |
| Coronary artery disease | 393 (10.9) | 335 (11.2) | 56 (10.3) | 0.61 |
| Congestive heart failure | 386 (10.7) | 303 (10.1) | 82 (15.1) | 0.001 |
| Liver disease | 227 (6.3) | 163 (5.4) | 64 (11.8) | <0.001 |
| Peripheral vascular disease | 223 (6.2) | 186 (6.2) | 35 (6.5) | 0.91 |
| Dialysis dependency | 127 (3.5) | 96 (3.2) | 31 (5.7) | 0.006 |
| Leukemia or lymphoma | 101 (2.8) | 76 (2.5) | 25 (4.6) | 0.012 |
| Clinical Frailty Scale | 4 (3–5) | 4 (3–5) | 5 (3–6) | <0.001 |
| Source of bacteremia |  |  |  | <0.001 |
| Urinary tract | 1523 (42.2) | 1326 (43.7) | 183 (33.5) |  |
| Intra-abdominal or hepatobiliary | 769 (18.8) | 561 (18.5) | 115 (21.0) |  |
| Lung | 469 (13.0) | 362 (11.9) | 103 (18.8) |  |
| Vascular catheter | 229 (6.3) | 180 (5.9) | 49 (9.0) |  |
| Skin and/or soft tissue | 187 (5.2) | 165 (5.4) | 18 (3.3) |  |
| Other | 67 (1.9) | 59 (1.9) | 8 (1.5) |  |
| Unknown | 454 (12.6) | 381 (12.6) | 71 (13.0) |  |
| Organism category |  |  |  | <0.001 |
| Enterobacterales | 2286 (63.4) | 1968 (64.9) | 304 (55.6) |  |
| Gram-positive | 626 (17.4) | 504 (16.6) | 113 (20.7) |  |
| Polymicrobial | 420 (11.6) | 346 (11.4) | 71 (13.0) |  |
| Non-fermenting Gram-negatives | 176 (4.9) | 131 (4.3) | 45 (8.2) |  |
| Anaerobes | 49 (1.4) | 41 (1.4) | 8 (1.5) |  |
| Others | 51 (1.4) | 44 (1.5) | 6 (1.1) |  |

Categorical variables are expressed as number (percentage) and continuous variables are expressed as median (interquartile range).

SOFA = Sequential Organ Failure Assessment; ICU = intensive care unit

1. **Table S3: Baseline characteristics of the study cohort, stratified by presence of persistent fever (>72 hours)**

| **Characteristic** | **No persistent fever**  **(n=3102)** | **Persistent fever**  **(n=506)** | ***p*-value** |
| --- | --- | --- | --- |
| Male sex | 1640 (52.9) | 282 (55.7) | 0.25 |
| Age, years | 71 (59–81) | 64 (52.3–73) | <0.001 |
| Baseline SOFA score | 4 (2–7) | 7 (4–10) | <0.001 |
| ICU admission at enrolment | 1548 (49.9) | 437 (86.4) | <0.001 |
| Mechanical ventilation at baseline | 530 (17.1) | 236 (46.6) | <0.001 |
| Diabetes mellitus | 995 (32.5) | 153 (31.0) | 0.54 |
| Solid organ cancer | 698 (22.8) | 84 (17.0) | 0.005 |
| Obesity | 546 (17.8) | 113 (22.9) | 0.009 |
| Arrhythmia | 487 (15.9) | 53 (10.7) | 0.004 |
| Immunosuppression | 262 (8.5) | 37 (7.5) | 0.48 |
| Chronic obstructive pulmonary disease | 353 (11.5) | 40 (8.1) | 0.030 |
| Renal insufficiency | 383 (12.5) | 52 (8.5) | 0.014 |
| Coronary artery disease | 346 (11.3) | 47 (9.5) | 0.28 |
| Congestive heart failure | 346 (11.3) | 40 (8.1) | 0.041 |
| Liver disease | 186 (6.1) | 41 (8.3) | 0.074 |
| Peripheral vascular disease | 204 (6.7) | 19 (3.8) | 0.022 |
| Dialysis dependency | 117 (3.8) | 10 (2.0) | 0.062 |
| Leukemia or lymphoma | 88 (2.9) | 13 (2.6) | 0.88 |
| Clinical Frailty Scale | 4 (3–5) | 3 (2–5) | <0.001 |
| Source of bacteremia |  |  | <0.001 |
| Urinary tract | 1366 (44.0) | 157 (31.0) |  |
| Intra-abdominal or hepatobiliary | 587 (18.9) | 92 (18.2) |  |
| Lung | 369 (11.9) | 100 (19.8) |  |
| Vascular catheter | 181 (5.8) | 48 (9.5) |  |
| Skin and/or soft tissue | 162 (5.2) | 25 (4.9) |  |
| Other | 58 (1.9) | 9 (1.8) |  |
| Unknown | 379 (12.2) | 75 (14.8) |  |
| Organism category |  |  | <0.001 |
| Enterobacterales | 2009 (64.8) | 277 (54.7) |  |
| Gram-positive | 518 (16.7) | 108 (21.3) |  |
| Polymicrobial | 338 (10.9) | 82 (16.2) |  |
| Non-fermenting Gram-negatives | 160 (5.2) | 16 (3.2) |  |
| Anaerobes | 34 (1.1) | 15 (3.0) |  |
| Others | 43 (1.4) | 8 (1.6) |  |

Categorical variables are expressed as number (percentage) and continuous variables are expressed as median (interquartile range).

SOFA = Sequential Organ Failure Assessment; ICU = intensive care unit

1.
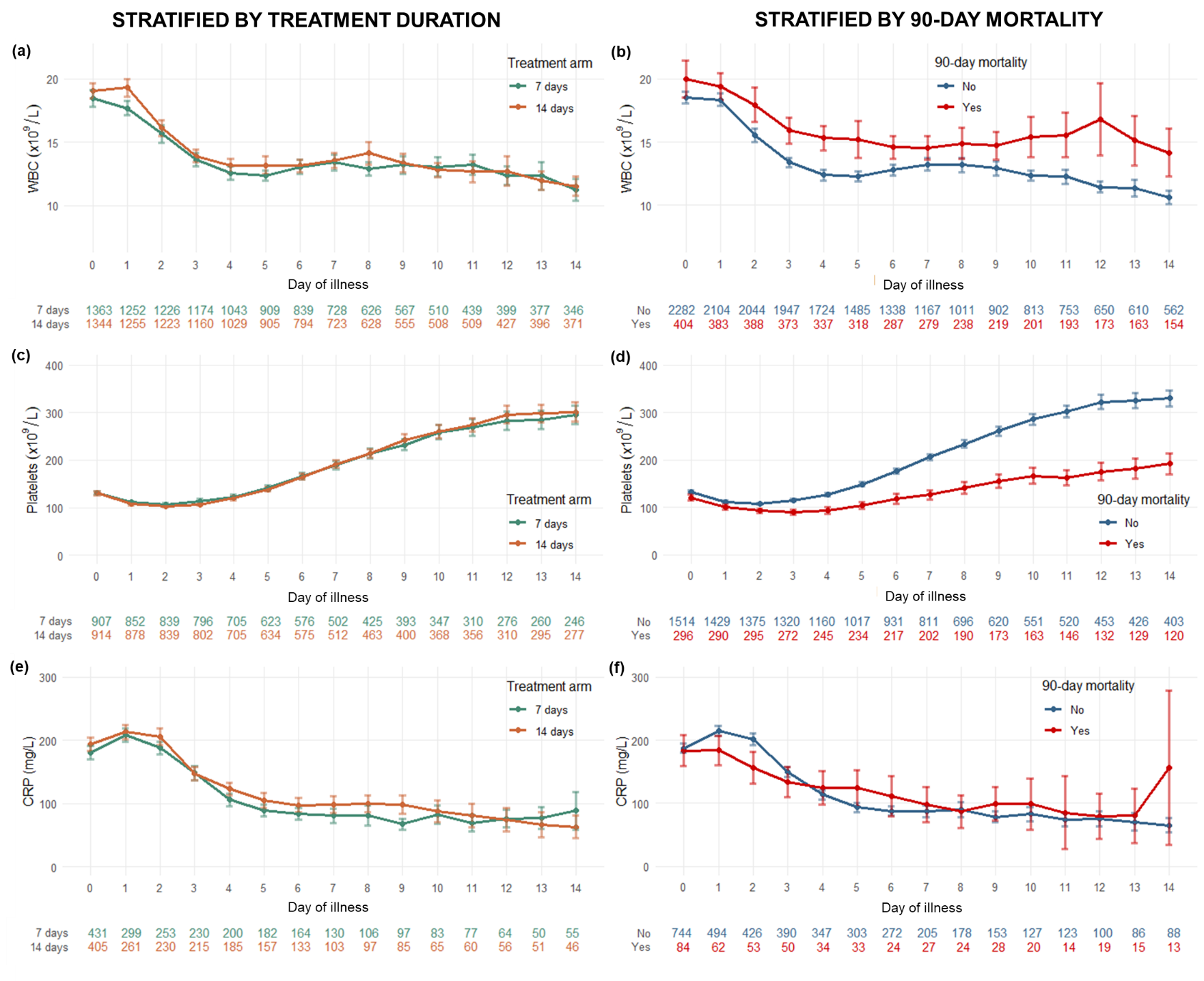
**Figure S1: Mean white blood cell count, platelet, and C-reactive protein count on each day of illness, excluding patients with normal baseline values.**

WBC = white blood cell count; CRP = C-reactive protein.

These graphs depict mean values for each biomarker on each day of illness, in the subset of patients with abnormal values at baseline (i.e., patients with normal values on day 0 of illness were excluded). Panels on the left show the subgroup stratified by treatment allocation group and panels on the right show the cohort stratified by primary outcome (90-day mortality). Corresponding numbers below each graph show the number of patients with available observations per subgroup for each day (e.g., on day 14 there were 346 patients in the 7-day arm and 371 patients in the 14-day arm who had available WBC readings).

1.
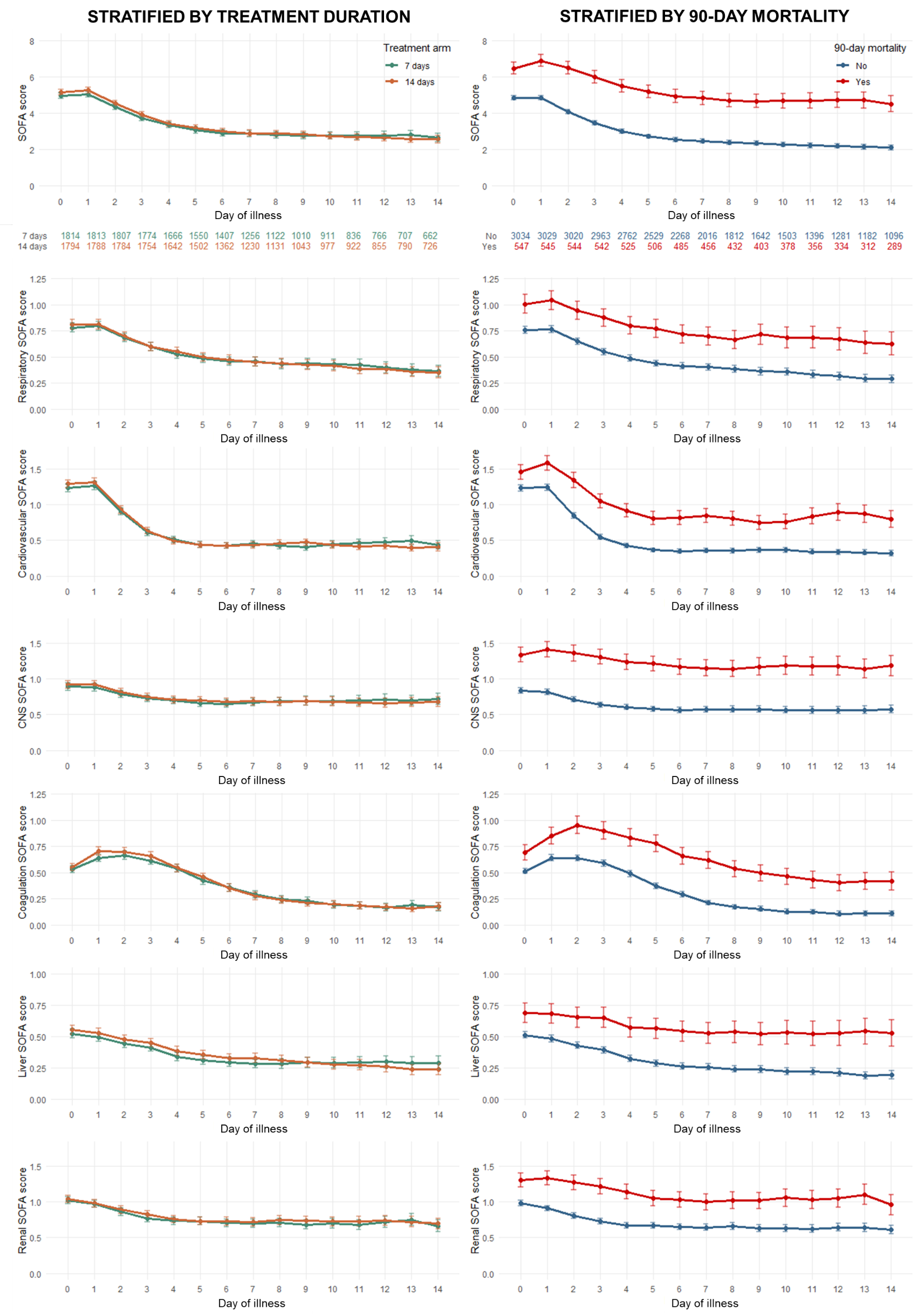
**Figure S2: Mean composite SOFA score and individual organ-specific SOFA score components on each day of illness.**
2. **
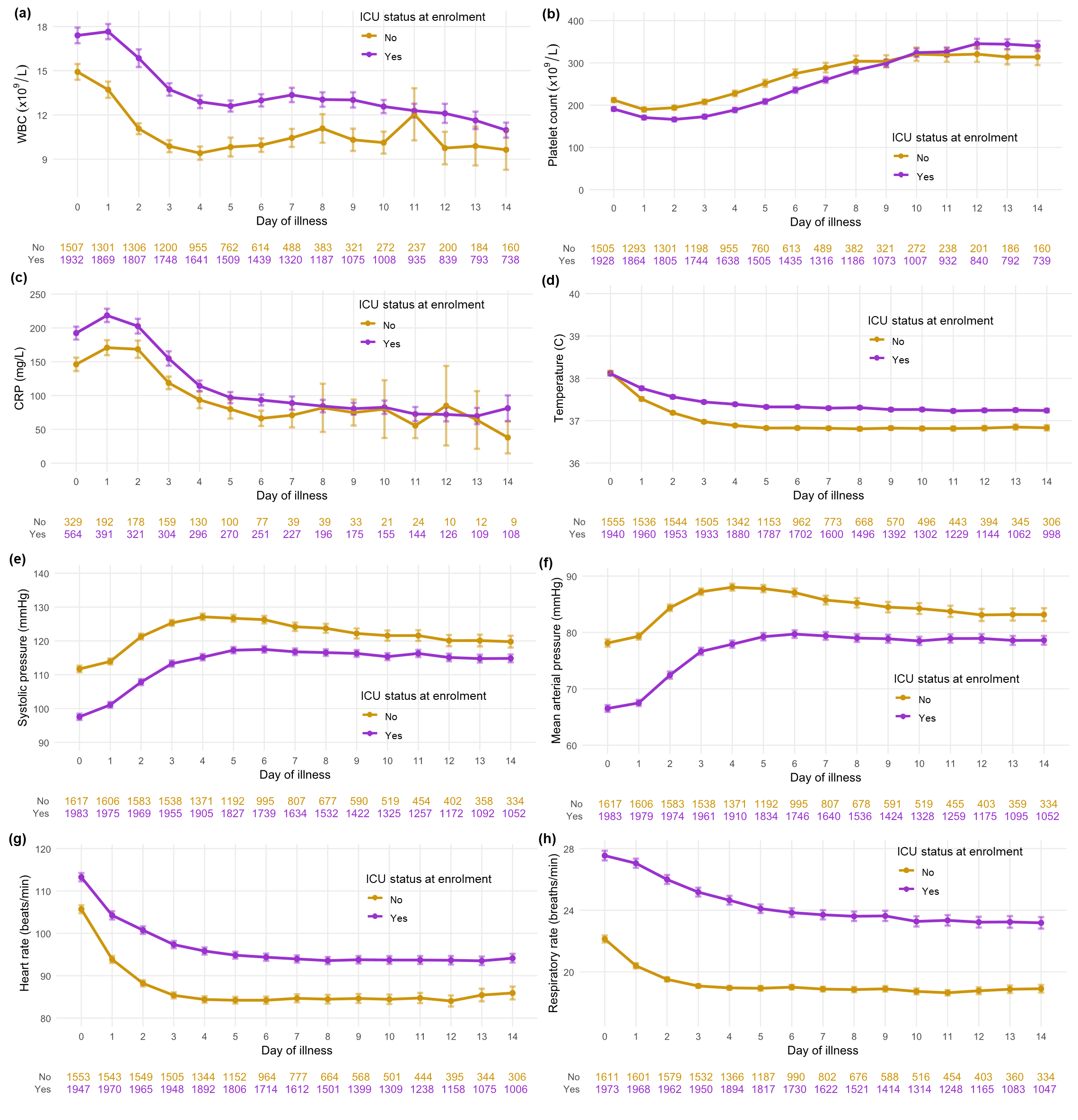
Figure S3: Mean values of eight clinical parameters by each day of illness, stratified by intensive care status at enrolment**

WBC = white blood cell count; CRP = C-reactive protein.

These graphs depict mean values for each clinical parameter on each day of illness, stratified by ICU status at enrolment. Corresponding numbers below each graph show the number of patients with available observations per subgroup for each day.

1.
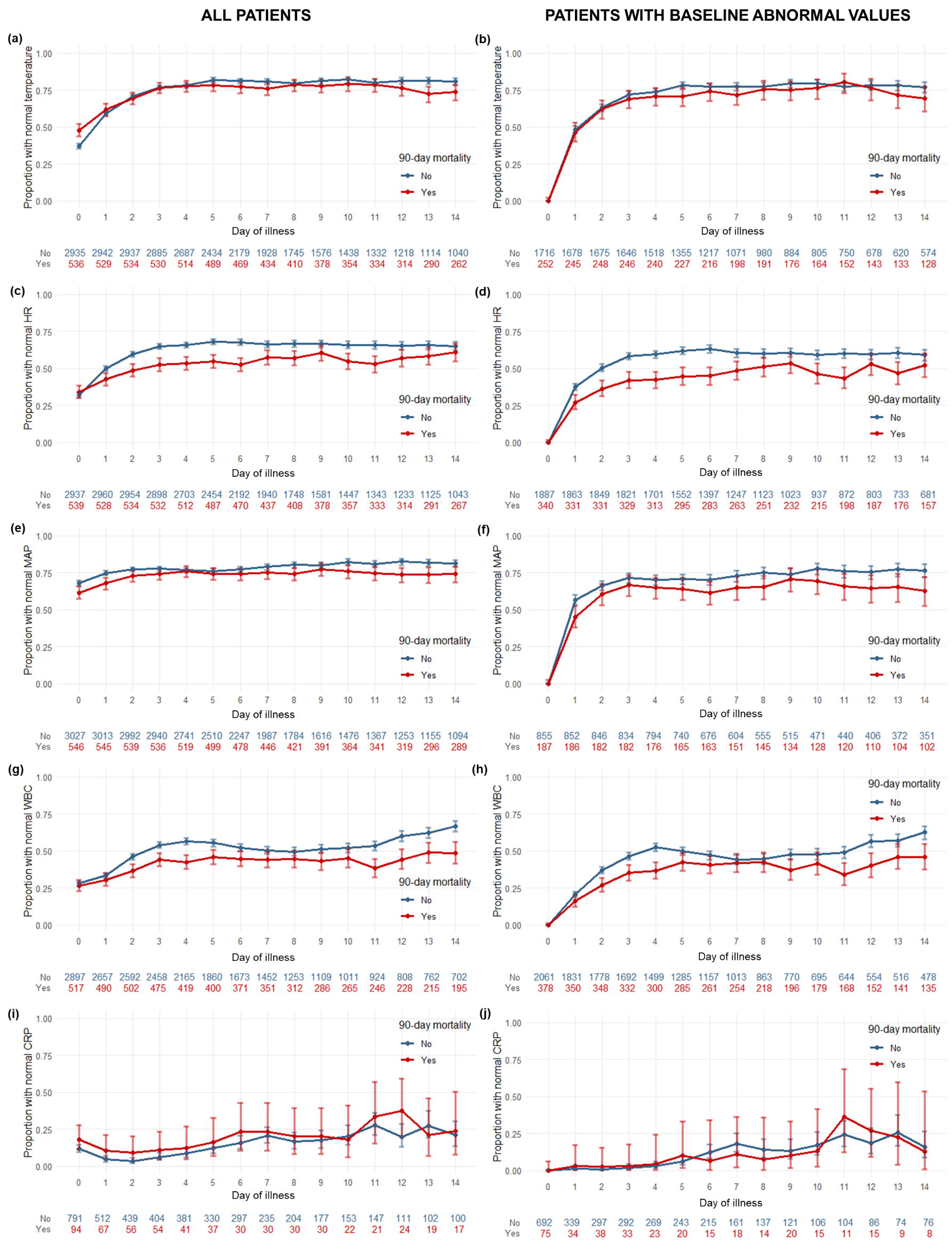
**Figure S4: Proportion of participants with normal temperature, heart rate, mean arterial pressure, white blood cell count, and C-reactive protein on each day of illness.**

HR = heart rate; MAP = mean arterial pressure; WBC = white blood cell count; CRP = C-reactive protein.

Normal ranges were defined as follows: temperature 36-38 °C, HR 60-100 beats/minute, MAP 60-100 mmHg, WBC 4-11 x10^9^/L, CRP <20 mg/L. The panels on the left include all patients with available data for each day. The panels on the right only include patients with abnormal values at baseline (i.e., excluding patients with normal values on day 0 of illness). Corresponding numbers below each graph show the number of patients with available observations per subgroup for each day (e.g., on day 14 there were 1040 patients who did not die within 90 days and 262 patients who died within 90 days who had available temperature readings).
